# Propensity score matching in estimating the effect of managerial education on academic planning behavior. Study design: a cross-sectional study

**DOI:** 10.1186/1472-6920-11-102

**Published:** 2011-12-08

**Authors:** Huy V Nguyen, An TM Dao, Dzung V Do

**Affiliations:** 1 Institute for Preventive Medicine and Public Health, Hanoi Medical University, 01 Ton That Tung Str, Dong Da Dist, Hanoi, Zip code: 10000, Vietnam; 2 Department of Biostatistics, Faculty of Public Health, Hochiminh University of Medicine and Pharmacy, 217 Hong Bang Str., District 5, Hochiminh City, Zip code: 70000, Vietnam

## Abstract

**Background:**

In many academic settings teaching a particular topic is applied to every student enrolled in the same academic year, it is a difficult task for researchers to design a randomized control group study. This research aimed to estimate the effect of teaching management and planning on increasing academic planning behavior (APB), using propensity score matching (PSM).

**Methods:**

In a cross-sectional survey utilizing a self-reported structured questionnaire on a systematic random sample of 421 students in Hanoi Medical University, one of the eight medical schools in Vietnam, this evaluation study adopted regression procedures to assess model fit, then PSM to create a matched control group in order to allow for evaluating the effect of management education.

**Results:**

The study showed both direct and indirect effects of the education on behavior. After PSM to adjust for the possible confounders to balance statistically two groups - with and without management education, there is statistically a significant difference in APB between these two groups, making a net difference of 18.60% (p < .05). The estimated 18.6 percentage point increase can be translated into the practice of APB by 670 students in the population. This number of academic planners can be attributed to a high recall of important management and planning education.

**Conclusions:**

The study provided theoretical as well as practical implications to guide the design of the education and evaluation of teaching.

## Background

Academic planning behavior (APB) may involve different tasks and skills of students - setting goals, planning activities, considering alternatives, monitoring and reflecting, as well as readjusting plans to meet progress rates [1,2]. A behavior as such plays a critical role in improving students' academic performance [1-4]. However, in various higher education settings APB has not been widely practiced among students; consequently, their academic achievement has been limited [5]. Teaching a topic on management in order to prepare or to direct students toward a well-planned study is therefore important if higher education programs are to be effective.

**Figure 1:**
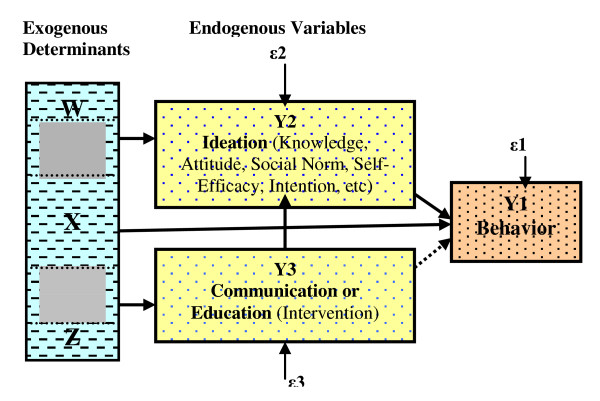
**A Model of Strategic Communication and Behavior Change**.

A theoretical framework for this study is based on a comprehensive conceptual model by Kincaid [6,7] (Figure 1) that has been adapted from a wide range of literature resources. Under this theory, the psychological influences including knowledge, attitude, social norm, intention, self-efficacy, and others can be combined as ideation. Specific communication interventions may be designed to influence only one or several types of psychological processes. All sorts of psychological processes are expected to affect behavior even if communication is designed to influence only one of them. Communication affects behavior indirectly by providing information that changes one or all of such processes. Exogenous determinants including demographic, socioeconomic and contextual characteristics affect endogenous variables - such as the exposure to the communication, ideation and behavior.

To review a higher education program, teachers and researchers would like to be able to estimate the effect of teaching a topic designed to change a behavior or to enhance academic performance. It has been well recognized among researchers and evaluators that calculating effectiveness is the most important part, but is sometimes the most challenging [8]. One can not claim a particular amount of behavior change without a causal attribution. A causal inference must be reached that attributes the net change in behavior to exposure to the intervention and not to other impacts or, worse yet, to changes that occurred before the intervention was implemented.

Because in many academic settings teaching a particular topic is applied to every student enrolled in the same academic year, it is a difficult task for researchers to design a randomized control group study. Many education and communication scholars have concluded therefore that it is virtually impossible to draw such a causal inference [9]. Without such a capability, how can we ever justify the influence of an education program?

In Vietnam, for medical students, to graduate as a medical doctor bachelor every student has to successfully pass, across six academic years - from 1 to 6, all topics, one of which is management and health management science which is offered by the Department of Health Organization and Management at the fifth academic year, usually during the annual fall (from August to October). Although several studies have been conducted, most looked at students' academic scores as main indicators to measure the effectiveness of teaching, while intermediate behaviors like planning skills which help students achieve higher academic progress, have remained under-researched. APB has become increasingly important as students were claimed under pressure in terms of time management due to a variety of theoretical and practical topics enrolled simultaneously in the same year. It is further pressure placed on students in terms of academic achievement due to a great volume of knowledge and skills to be learned within universities. To assess the impact of an education program, researchers and evaluators may adopt different methods. Among these, PSM is highly recommended as it is one of the strong statistical techniques [6-8,10,11] as to be discussed. This method can help to reduce selection bias as it allows for quasi-experimental contrasts between students in receiving "treatment" and "control" groups based on their observed characteristics. Proper use of PSM should also allow for rigorously derived and relatively unbiased estimates of communication's or education's effects on participants' behavior [12]. Because of its ability to reduce selection bias, PSM has become increasingly used in the fields of education [13,14], communication [6-8,10], medicine and epidemiology [15], policy evaluation [16], economics [17], psychology [18]. Although commonly applied in diverse disciplines in many contexts, too little has been achieved in measuring APB as an intermediate outcome that helps to enhance students' academic progress.

*The purpose of this study is to use propensity score matching (PSM) to estimate the impact of teaching management on APB among medical students in a Vietnamese medical education context*.

## Methods

### Study design and setting

An evaluation study was conducted with a cross-sectional sample survey using a self-administered structured questionnaire. The study was carried out in Hanoi Medical University (HMU), located in northern Vietnam. This is one of the eight medical universities in the country. Similar to all eight medical universities, HMU offers teaching a managerial topic with the same contents, one of which is management and planning science designed to help students develop planning knowledge and skills, which is helpful to their current study and their future health care career.

### Participants and sampling procedure

A representative sample survey of 421 students across academic years 1-6 was conducted within November to December 2009. This sample was obtained based on a systematic random technique for the survey given a sampling frame of 3145 students, a total population size of undergraduate students from the first to sixth year. The sampling frame was provided by the Director of the HMU Undergraduate Training Department. Following a random start, 421 students were selected, using a fixed interval of 7.

### Data collection

The material for data collection was a self-administered structured questionnaire. It included 4 sections, first asked students about their individual and social characteristics, second the level of access to the topic of management science, third knowledge, attitudes and self-efficacy of academic planning and finally APB during the past and current year.

As a procedure, the study was first approved by the HMU Undergraduate Training Department, then all selected students were informed of the study objectives and contents. 95% of the sample gave their informed consent to participate; because 5% refused the study, we used the same sampling strategy to approach more students until a full size of 421 students was reached. Both male and female medical students were surveyed, using the anonymous self-reported manner. Each student completed a confidential questionnaire for 15 to 20 minutes either before or after a lecture he or she attended as well as after permissions received from teachers. The Institutional Review Board (IRB) of the HMU has not required a formal ethical approval for the survey.

This study was informed by a pilot survey to validate the instrument. The pilot showed that the instrument was technically feasible for the main survey.

### Measurement

Exposure to management education can be measured as a state whether a student has learnt management topic. However, a good method of communication or education evaluation requires a valid, reliable measure of recall of key contents at the interval level of measurement. In this study, therefore, an open-ended question was added to ask students to remember and list all possible contents of lessons they have learned. To transfer textual data into standard contents, a principal investigator from the topic of management and health management, who knew well about the topic and its contents, closely looked at all textual responses, then classified if each of them would go under the standard contents of the topic. In total 8 standard contents were identified. The above question formed a continuous scale measuring the level of recall that ranged from 0 to 8 contents with a median of 4. Recall of at least one content was 90%. To simplify this measurement and accommodate the logistic regression and propensity score analysis, the scale was classified into 1 and 0 (higher versus lower recall).

Management and planning knowledge was measured by 8 true/false/don't know items such as "*a good academic planning includes setting goals and considers timeline frame and resources*". Scoring the knowledge scale was accomplished by dichotomizing each item into a value of 1 (correct) and 0 (incorrect or don't know) and then summing the item values to form a composite score with higher scores reflecting better knowledge (Cronbach's alpha = .50; mean score = 6.32, SD = 1.96). Attitude toward academic planning was measured with seven 5-point semantic scale (*bad-good*) from 1 (negative evaluation) to 5 (positive evaluation) such as "*how good or bad would it be if you talked about academic planning for each topic with your friends every year?*" A composite score was obtained by summing responses to items with higher composite scores indicating higher levels of attitudes (Cronbach's alpha = .55; mean score = 16.26, SD = 3.76). Social norm toward academic planning was assessed with seven 5-point semantic scale (*untrue-true*) from 1 (negative evaluation) to 5 (positive evaluation) such as "*Most people - family, teachers and close friends - who are important to you think you should talk about academic planning with your friends every year*?" A composite score was obtained by summing responses to items with higher composite scores indicating higher level of social norm (Cronbach's alpha = .85; mean score = 13.57, SD = 4.30). Intentions for academic planning are measured by asking students to rate on a 5-point semantic scale ranging from *very unlikely *(1) to *very likely *(5) such as "*during the next few months, you intend to talk about academic planning with your friends for each topic?*". A composite score was formed by summing responses to items with higher scores indicating higher levels of intentions (Cronbach's alpha = .83; mean score = 15.62, SD = 5.17). Self-efficacy of academic planning was assessed with seven items tapping perceived difficulty of academic planning on a 5-point semantic scale from *very hard *(1) to *very easy *(5) such as "*how hard would it be for you to advice or persuade your friends to make academic planning?*" A composite score was obtained by summing responses to items with higher scores reflecting higher levels of self-efficacy (Cronbach's alpha = .63; mean score = 18.61, SD = 5.10). These five related sub-constructs representing the cognitive and social interaction component of ideation were used to construct the measures of ideation. For the logistic regression analysis, using a cut-off of 50% the measures were split into 1 and 0, corresponding to higher and lower levels of ideation.

Past year and current APB were combined to construct a continuum of behavior with the following five scale values: *0 = never, 1 = rarely, 2 = occasionally, 3 = usually*, and *4 = always *(Cronbach's alpha = 0.70). Combining these two items into a single outcome variable has two advantages in that it makes the measurement more valid and reliable as well as allows the analysis of the impact of education on students' APB. The levels of APB met the minimal requirements of order with respect to ideation with the support from [19] one-sided test of significance performed with *p *= .01. This level of probability indicated the rejection of the null hypothesis of equality of levels and supporting the alternative hypothesis of order. To make logistic regression analysis and estimation of the impact possible, the scale of the single APB was classified as 1 and 0 reflecting more and less frequent level of APB.

Socioeconomic status was measured by means of living total money received from family categorized into two levels - higher and lower socioeconomic status. Age was measured by number of years categorized into higher and lower groups. Level of university education was measured by ordinal number of academic years categorized into seniority (three final academic years) and juniority (three first academic years). Origin of permanent residence was classified into urban and rural area. Demographic characteristics including gender, ethnics and religion were also included. Further, prior research and literature has indicated that a student's father and/or mother occupation - called parental occupation (white vs. blue collar) predicts his or her study behaviors and academic achievement [20-22]. To maximally reduce the potential for selection bias, we included these covariates in the model predicting propensity to receive education [23].

### Statistical analysis

The software program "STATA version 10.0" was applied to all the following statistical procedures.

#### Simple proportion differences

We used Chi-square tests to determine whether proportion differences in APB of students receiving and not receiving management and planning education were statistically significant. We used a *p *value of .05 for these analyses. We considered the results obtained from Chi-square tests of these proportion differences as a "benchmark" for the results obtained from analyses using PSM as referred to below.

#### Logistic regression modeling

The model of direct and indirect effects used for this analysis requires three equations, one for each endogenous variable: APB, ideation, and recall of the planning contents educated. Each of such equations is presented as follows:

$$\begin{array}{c} {\text{Y}}_{\text{1i}}=\beta_{0}+\beta_{1}{\text{Y}}_{\text{2i}}+\beta_{2}{\text{Y}}_{\text{3i}}+\beta_{3}{\text{X}}_{\text{i}}+\;\mu_{\text{i}}\\ {\text{Y}}_{\text{2i}}=\gamma_{0}+\gamma_{1}{\text{Y}}_{\text{3i}}+\gamma_{2}{\text{W}}_{\text{i}}+\;{\text{v}}_{\text{i}}\\ {\text{Y}}_{\text{3i}}=\sigma_{0}+\sigma_{1}{\text{Z}}_{\text{i}}+\;\xi_{\text{i}} \end{array}$$

Where Y_1i _is APB for subject i, Y_2i _is ideation, Y_3i _represents exposure to management and planning education for subject i, X_i_, Wi and Zi are matrices of exogenous socioeconomic and demographic control variables, the three β, two γ and one σ coefficients are parameters to be estimated from the data, and μ_i_, vi, and ξi are the disturbance (residual) terms. Because APB, ideation, and exposure are measured with a binary scale, logistic regression is used to estimate the parameters of the equation. The differentiation among the X, Z, and W matrix of exogenous control variables indicates that each endogenous variable should be determined by exogenous variables not included in the other two equations. However, some overlap of exogenous variables can be acceptable, but each endogenous variable must have at least one exogenous control variable that is excluded from the equations for all other endogenous variables [24]. The arrows indicate the hypothesized direction of influence and effects, that the three error terms are uncorrelated, and that there is no third unobserved variable that accounts for any of the hypothesized relationships. In examining the model fit of these three equations, tests for endogeneity are used to determine if there are any unobserved variables responsible for the observed relationship between exposure and the outcome, and if the relationship is reciprocal. The thread of endogeneity can be ruled out if the disturbance terms, ε2 or vi and ε3 or ξi, are statistically uncorrelated as reflected as rho and if they are not statistically significantly when added to the equations for behavior ad ideation, respectively. If the coefficient of the estimated error term (ε2, ε3) is not significantly different from 0, one would accept the null hypothesis that the suspected endogenous variable is an exogenous variable, and that there is no unobserved variable affecting on the outcome y3 for the treatment (education exposure); and, therefore, y3 can be regarded as exogenous, and simple regression can be adopted [8].

#### Creating the matched sample using propensity score matching

A propensity score is the probability of being exposed to a treatment or an intervention given a set of observed covariates, *X*. The method as this was developed as a means to balance the treatment and control units so that a direct comparison would make a valid conclusion. The technique was found robust as recently in practice it was difficult, if not possible, to match on more than 2 variables. PSM therefore makes it feasible to create an unexposed comparison group that is statistically equivalent on the average to subjects who are exposed to the treatment [25,26]. For research survey, a single score for matching is generated using statistically regressing exposure on all of the variables that determine exposure and also may be related to the outcome variable [8].

This technique requires a two-stage process. Stage 1 involves the use of a logistic or probit regression model to calculate all respondents' propensity for experiencing a treatment of interest, in this case, receiving management and planning education (MPE). The propensity score is defined as follows [25]:

p(T)≡Pr2=E{T∣S}

Where p(T) is the propensity to be placed into MPE, T indicates that a student did or did not receive MPE, and S is a vector of covariates influencing whether the student did or did not receive MPE. In Stage 2, we used the estimated propensity scores obtained in Stage 1 to match students who did and did not receive MPE. To obtain a full sample, we used stratification matching which uses all treatment and control cases. Using the STATA 10.0 "atts" command, the full range of sample members' propensity scores is divided into propensity score strata, or blocks, each of which includes treatment and control cases with the same or nearly the same propensities for receiving the treatment. The number of appropriate strata depends on the number necessary to gain a balanced propensity score. Within each of these strata, the ATT is calculated, and then the ATT's across strata are averaged to produce a final ATT.

## Results

### Sample characteristics

In total, 421 undergraduate students were asked to participate in the study. The proportions were fairly evenly distributed by gender (male vs. female, each with ~50%), academic years (from year 1 to year 6, each ~16.5%), and place of permanent residence (urban vs. rural, each ~ 50%). The mean age of all participants was 21 years (range 18-27 years: SD = 1.89). The distribution of monthly stipend from family for living and studying was VND1.36 million (range .3-10: SD = .72; US$1 = VND19,000). The main differences were in ethnicity (most were the Kinh, a major ethnic group in Vietnam), religion (most were the followers of Buddhism, ancestor worship and non-religious), and parental occupation, with white collars accounting for almost 70% of the total.

### Simple proportion differences

Table 1 compares APB proportions between the study's subsamples of those who exposed to and did not expose to MPE. There were unadjusted proportion differences in all categories of APB between these two groups. Results indicate that students receiving MPE displayed higher proportions of APB. Overall, students who had a higher recall of MPE contents showed a higher proportion of APB compared to those with a lower recall (71.51% vs. 42.28%; with a relative gain of 29.23 percentage points). This difference is statistically significant at the *p *< .001 level.

**Table 1:** Dependent variable proportions for MPE students and non-MPE students

Dependent Variable (APB)	Exposed Students n(%)	Non-Exposed Students n(%)	P (χ^2^)
	**Enrolled in MPE (n = 146)**	**Non-Enrolled in MPE (n = 275)**	
APB during the past year APB during the current year	65(44.52) 69(47.26)	85(30.91) 99(36.00)	** *
	**Exposed to 50% or more of all MPE sessions (n = 123)**	**Exposed to just less than 50% of all MPE sessions (n = 298)**	
APB during the past year APB during the current year	57(46.34) 61(49.59)	93(31.21) 107(35.91)	** **
	**Higher recall (n = 123)**	**Lower recall (n = 298)**	

**Composite APB**	**88(71.51)**	**126(42.28)**	***

*p < .05; **p < .01: ***p < .001

### Logistic regression modeling for predictors of recall, ideation and APB

The results of the logistic regression analysis using probit procedure are shown in model 1 of Table 2. Descriptive statistics for each variable are reported in the second column. The equation for recall, ideation, and APB were identified with 9 exogenous variables. Results of the exclusion tests are reported at the bottom of the column for each equation. No statistically significant effects of exclusion were found. The proportion of the variance explained by the equation for recall, ideation, and APB was .27, .22, and .32, respectively. As expected, the best predictor of APB were ideation toward APB (.75, p < .001). Recall of MPE contents also had a direct, significant effect on APB (.57, p < .05) as well as an indirect effect through its effect on ideation (.50, < .05). These results indicate that ideation is a mediating (intervening) factor between MPE recall and APB and hence one of the causal pathways by which education has an effect. This indirect effect is what the content of the education was promoted to achieve, which confirms the theory as well as the empirical impact. The direct effect of MPE recall on ideation was statistically significant after controlling for the effect of a number of demographic and socioeconomic variables.

**Table 2:** Results of the structural equation model and propensity score analysis of APB

Variables	Description % or mean (range)	Model 1 Logistic regression (probit procedure)			Model 2 Propensity Analysis	
		
		Recall (Probit coef.)	Ideation (Probit coef.)	APB (Probit coef.)	Recall (Probit coef.)	APB (Probit coef.)
Age (older vs. younger)	21.04 (18-27)	1.22***	0.20***	-	1.22***	-
Gender (female vs. male)	53.00	0.82***	0.17	-	0.80***	-
Parental occupation (white vs. blue collar)	41.09	0.07	0.29*	-	0.06	-
Place of permanent residence (urban vs. rural)	47.27	0.19*	-	-	0.18	-
Ideation (higher vs. lower)	18.05			0.75***		0.75*
Recall (higher vs. lower)	29.22		0.50*	0.57*		0.47*
Academic year (senior vs. junior)	51.54	-	-	0.19*	-	0.10
Living stipend level (million Vietnamese Dong)	1.36 (0.3-10)	0.14	-	0.11	0.15	0.11
Religion (yes vs. no)	2.14	-	0.60	-	-	-
Number of cases	421	421	421	421	421	421
Variance explained (adjusted R^2^)		0.27	0.22	0.32	.20	0.12
Exclusion test (model fitness) (*p*)^1^		NS	NS	NS	NS	NS

Test for endogeneity - Biprobit rho for Model 2: 0.07 (-0.30-0.43)

rho-based *p: *Chi^2 ^= 0.1378; p > 0.710

Statistical significance: *p < .05; **p < .01; ***p < .001; NS = not significant

Exclusion of a variable for model identification is indicated by (-).

^1^The likelihood ratio test was used for binary dependent variables.

The effects of the exogenous control variables are also theoretically relevant. Recall of the MPE was related to age, gender and place of permanent residence. Ideation about APB was related to age and parental occupation. APB was related to academic year. There is no reason why religion would be related to recall and APB, so it was excluded from those equations. However, religion was related to ideation (including attitudes, social norms, and intentions), albeit not significant. Because age, gender, parental occupation, and place of permanent residence may be neither as well as would nor expected to be, related to APB, so they were excluded from the APB. Theoretically, it was found that place of permanent residence would not influence ideation, thereby being excluded.

### Propensity score analysis

The results underlying the propensity score analysis are reported in the second model presented in Table 2. Propensity score analysis was done for the level of recall (high versus low/no recall). To test for endogeneity and support for the assumption of strong ignorability, we treated the problem as a two-equation system. The results showed that high recall of MPE contents had a statistically significant direct effect on APB (.47, p < .05) after controlling for the remaining variables in the model. Biprobit analysis of these two equations was conducted to test for the endogeneity of level of recall in the APB equation. The correlation of the error terms was not statistically significant (rho = .07, p > .05), indicating that MPE recall may be considered as exogenous in the equation of APB. When the error term from the equation for recall was added to this equation, it was not statistically significant as well. This suggests that ordinary probit regression may be used to estimate the direct effects of high recall of MPE contents and that the potential effects of omitted or unobserved variables may be ignored. The result of the propensity score analysis as informed from these equations is therefore expected to approximate what we would expect from a randomized control group design.

Table 3 shows the results of the stratification of propensity score. We used stratum-based PSM in order to maintain the full sample design and yield results based on the same cases as the SEM analysis. The propensity score - probability of high recall - ranged from .002 to .999 with a mean of .29 (SD = .36). The continuous score was stratified into 6 balanced subgroups within which there were no statistically significant differences in the propensity between those with high recall (treatment group) and those with no/low recall (matched control group). There were also no statistically significant differences for any of the 7 variables used in the regression within any of the six subgroups (42 tests). Results of the propensity score analysis showed that 71.51% of participants in the treatment group (with high recall) practiced academic planning after the MPE compared to 52.65% of participants in the matched control group (with low/no recall), a statistically significant difference of 18.60 percentage points (Z test-based *P *< .05).

**Table 3:** Balance of the propensity scores for MPE exposure: Results from PSM

Stratum	Non-Exposed to MPE (A) N	Exposed to MPE (B) N	Total	Net Difference (B-A) Using "Atts" Command
1 (range of pscore = 0 - ~0.1)#	221	0	221	
2 (range of pscore = 0.1 - ~0.2)#	39	6	45	
3 (range of pscore = 0.2 - ~0.3)#	7	4	11	
4 (range of pscore = 0.3 - ~0.4)#	19	26	45	18.6% (p < 0.05)
5 (range of pscore = 0.4 - ~0.5)#	7	20	27	
6 (range of pscore = 0.5 - ~1)#	5	67	72	

**Total**	**298**	**123**	**421**	

Average propensity score = .29; SD = .36; Range = .002 - .999

# pscore statistics indicate that there is no statistical difference between A and B within each stratum (p > .05), meaning that propensity scores balanced at 6 strata

**Figure 2:**
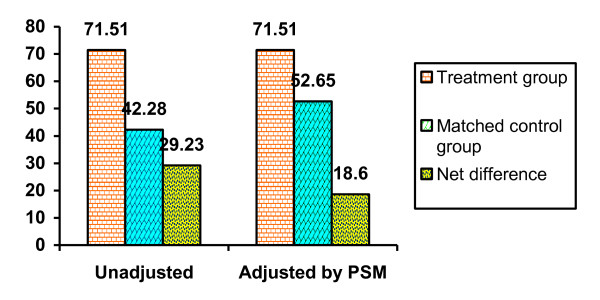
**Comparison of the unadjusted increase in APB to the increase adjusted by PSM (N = 421)**.

Figure 2 presents the simple bar graph comparing the results of the analysis between unadjusted and adjusted by PSM. The unadjusted difference in APB between the treatment group and matched control group was 29.23 percentage points (71.51% minus 42.28%). The propensity score results were based on the weighted sum of differences in APB among the balanced, statistically equivalent subgroups. The adjusted difference between the treatment group and matched control group was 18.6 percentage points (71.51% minus 52.65%). By statistically balancing the treatment group (high recall) and the matched control group (low/no recall) based on the propensity scores and all the variables used to construct it, the estimate of impact declines 10.63 percentage points. The estimated 18.6 percentage point increase in APB as an impact of the education can be translated into the practice of APB by 670 students in the population (this calculation was based on the total population of 3145 undergraduate students). This number of academic planners can be attributed to a high recall of the education.

## Discussion

An important feature of a quality education program is a regular review of its curricula in order to improve it [27]. But, before so doing, teachers and researchers would like to see what impact their designed and delivered education program has had on changing students' learning behavior or achievement. Therefore, this study aimed to estimate the impact of teaching a topic in a medical education institution in Vietnam on students' learning behavior. In particular we sought to quantify the effects of the designed and delivered management and planning education on students' academic planning behavior. We did so using a fairly large sample of such students and methods that would help greatly reduce selection bias. Because we could not randomly assign students to receive or not receive MPE, we used propensity score matching techniques to contrast the behavior of students who did and who did not receive this education but who had been matched on a variety of observed background characteristics. We also used some other important analyses such as structural equation modeling to identify the model of multivariate effect.

This study provided a combination of tests - logistic regression, structural equation modeling and propensity score matching - of the effect of the MPE on ideation and on APB. In Vietnam, the MPE is delivered for the fifth academic year medical students, usually during the annual fall (from August to October). Afterwards we conducted a data collection. With data from a representative sample survey of the students, propensity score matching was able to provide a valid counter-factual condition - the matched control group and therefore an unbiased estimate of a percentage point increase in academic planning behavior that would not have happened without the MPE. Specifically, we found that students who had a higher recall of MPE contents displayed a higher proportion of academic planning behavior compared to those with a low and no recall (71.51% vs. 52.65%, p < .05) for a net increase of 18.6 percentage points. The MPE reached most of students at the fifth and sixth years as many sessions of this topic were compulsory to students when enrolled in it. An average of 2.52 MPE contents out of 8 were recalled. Bivariate analysis showed that content recall was significantly related to ideation and APB, and ideation was also strongly related to APB. More important, the multivariate analysis revealed that the level of ideation and level of MPE recall was also related to APB.

The after-only, cross-sectional regression analysis without ideation showed a significant direct effect of the communication or education campaign on the adoption of a behavior [8,10] after controlling for socioeconomic variables. This is as much as many studies of mass media impacts are able to do [6-8,10,11]. When the composite measure of ideation was added to the after-only regression equation, the direct effect of the communication and/or education was no longer statistically significant because of the strong effect of ideation [6-8,10]. However, we found in our study that the after-only regression analysis of ideation showed that the content recall had the strongest effect on ideation and the regression analysis of APB showed that the recall had the second strongest effect on APB after the ideation. Therefore, the results confirmed the indirect effects of the education, but also its direct effect on behavior, emphasized the role of mediating effect of ideation, and provided support for the theoretical model that guided the design of the education or intervention and the evaluation of the results.

The structural equation modeling could provide support for a causal inference. The three equations (represented in model 1, Table 2) controlled for potentially confounding (socioeconomic) variables that might affect behavior. After controlling for these variables, the MPE content recall had a significant effect on both ideation and APB, and ideation had a significant effect on APB. The potential effect of unobserved variables (not in the equations) and the reciprocal effect of behavior on ideation and recall were ruled out by the statistical tests for endogeneity. The only criterion missing for a causal inference was a counterfactual condition which could have been provided only in a controlled experimental design. In our study, the counterfactual condition was made by propensity score matching in order to create a matched controlled group so the comparison of net difference would be possible. However, acceptance of such a causal inference for MPE and ideation on APB does not necessarily mean that other causes were not also operating. Students would approach or may be exposed to other sources of MPE such as internet, library or others. But at least in this study we could argue that the effect on APB would be as a result of the MPE per se which was designed and delivered by the Department of Health Organization and Management because we measured the recall of the key contents taught only at the university. Comparing between the treatment group and matched control group, a 18.6 percentage point increase in APB after the education delivered may sound small. However, because the sample of 421 represents a population of 3,145 students, the actual net increase in the number of students practicing APB is estimated to be 670.

Although many efforts have been made, our study can have several limitations. First, because many sessions of MPE - lectures and practicum - were compulsory, there would be an efficient way to reach students as well as MPE. In this situation, the risk of falsely rejecting an effective treatment (Type II error) appears to be greater than the risk of falsely rejecting the null hypothesis (Type I error) [28]. Further, due to the nature of self-reported research design, recall bias would be inherent. Some students, perhaps because of their self-esteem, could over- or under-estimate their behavioral responses. However, as this study designed a survey on a fairly large, representative sample with anonymous and confidential commitment, it would partly reduce such a bias. Also, since students self-reported their academic planning behavior, further studies should combine self-reporting of APB and observation or collection of actual plans (study notes, schedules, timetable, etc) of students. Moreover, as a cross-sectional study, this design may preclude the order of causality; therefore, a longitudinal study is needed to address this concern.

Interpretations of this study provide a theoretical as well as a practical implication. Consistent with the literature, our study informed the indirect effect of the education or communication (message recall) on behavior or the intervening role of ideation between education and behavior in the theoretical framework. At the same time, we also indicated the direct effect of the education on behavior. This suggests that designs of education evaluation should include ideation in addition to the education or recall in order to obtain a holistic theoretical model to support research. Another practical concern is: Should lecturers or institution leaders take action based on this conclusion? Should they review curricula and teaching using structural equation modeling and propensity score matching? The results of this study suggest that such a technique, a reliable, but a still neglected method of research and evaluation in many educational contexts, should be rolled out to other topics of education evaluation.

## Conclusion

Adopting PSM has made it possible to create a matched control group, allowing for estimating the net impact of managerial education on APB.

The results confirmed the indirect effect of the education on behavior or the intervening role of ideation between education and behavior. The direct effect of the education on behavior was also identified.

PSM adjusting for the possible confounders, there is a statistically significant difference in APB between students who did and did not recall management contents.

The study provided a theoretical as well as a practical implication to guide the design of the education and the evaluation of teaching and/or curriculum

## Abbreviations

APB: Academic planning behavior; HMU: Hanoi Medical University; IRB: Institutional Review Board; MPE: Management and planning education.

## Competing interests

The authors declare that they have no competing interests.

## Authors' contributions

HVN developed a concept protocol, analysed data, drafted and revised the manuscript. ATMD drafted and revised the manuscript. DVD reviewed, revised and edited the manuscript. All authors read and approved the final manuscript.

## Pre-publication history

The pre-publication history for this paper can be accessed here:

http://www.biomedcentral.com/1472-6920/11/102/prepub

## References

[B1] Eilam B Aharon I Students' planning in the process of self- evaluated learning Contemporary Educational Psychology 2003 28 304 334 10.1016/S0361-476X(02)00042-5

[B2] Study Skills Self Help Information http://www.ucc.vt.edu/stdyhlp.html

[B3] Zimmerman BJ Schunk DH Self- Evaluated Learning and Academic Achievement: Theoretical Prospectives 2001 Mahwah, USA: Lawrence Erlbaunm Associate

[B4] Schunk DH Commentary on self-regulation in school contexts Learning and Instruction 2005 15 173 177 10.1016/j.learninstruc.2005.04.013

[B5] Norman GR Schidmit HG The psychological basis of problem-based learning: a review of the evidence Academic Medicine 1992 67 9 557 565 10.1097/00001888-199209000-00002 1520409

[B6] Kincaid DL Advanced Research Methods in Evaluating Communication and Publich Health Programs Using Structural Equation Modelling (SEM) and Propensity Score Procedures 2008 Hanoi, Vietnam: Centre for Community Health Research and Development (CCRD)

[B7] Kincaid DL Data Analysis Using Logistic Regression and Structural Equation Modelling (SEM) 2009 Hanoi, Vietnam: Centre for Community Health Research and Development (CCRD)

[B8] Kincaid DL Do MP Multivariate causal attribution and cost-effectiveness of a national mass media campaign in the Philippines Health Communication 2006 11 69 90 10.1080/10810730600974522 17148100

[B9] Kincaid DL Phuong DM Multivariate causal attribution and cost-effectiveness of a national mass media campaign in the Philippines Journal of Health Communication 2006 11 69 90 10.1080/10810730600974522 17148100

[B10] Kincaid DL Mass media, ideation, and behavior: A longitudinal analysis of contraceptive change in the Philippines Communication Research 2000 27 6 723 763 10.1177/009365000027006003

[B11] Kincaid DL Coleman P Pham V The Impact of AIDS Communication Programs on HIV Prevention Behavior and Living with HIV/AIDS: National HIV/AIDS Communication Survey 2006 2006

[B12] Dehejia RH Wahba S Causal effects in nonexperimental studies: Reevaluating the evaluation of training programs The Amerian Statistical Association 1999 94 1053 1062 10.2307/2669919

[B13] Rosenbaum PR Dropping out of high school in the United States: An observational study Journal of Educational Statistics 1986 11 207 224

[B14] Schneider B Carnoy M Kilpatrick J Schmitdt WH Shavelson RJ Estimating Causal Effects Using Experimental and Observational Designs 2007 Washington, DC: American Educational Research Associaton

[B15] Stone RA Obrosky DS Singer DE Kapoor WN Fine MJ The PORT Investigators: Propensity score adjustment for pretreatment differences between hospitalized and ambulatory patients with community-acquired pneumonia Journal of Medical Care 1995 33 56 66 7723462

[B16] Harknett K Does receiving an earnings supplement affect union formation? Estimating effects for program participants using propensity score matching Evaluation Review 2006 30 741 778 10.1177/0193841X06293411 17093107

[B17] Lechner M Program heterogeneity and propensity score matching: An application to the evaluation of active labor market policies Review of Economics and Statistics 2002 84 205 220 10.1162/003465302317411488

[B18] Green KM Ensminger ME Adult social behavioral effects of heavy adolescent marijuana use among African Americans Developmental Psychology 2006 42 1168 1178 17087550 10.1037/0012-1649.42.6.1168 PMC5784847

[B19] Jonckheere AR A distribution-free k-sample test against ordered alternatives Biometrika 1954 41 1-2 133 145 10.1093/biomet/41.1-2.133

[B20] Keeves JP Introduction to Statistics and Data Processing 2004 Flinders University, Australia: Department of Education

[B21] Keeves JP Multilevel and Multivariate Analysis 2005 Flinders University, Australia: Department of Education

[B22] Livaditis M Zaphiriadis K Samakouri M Tellidou C Tzavaras C Xenitidis K Gender differences, family and psychological factors affecting school performance in Greek secondary school students Educational Psychology 2003 23 2 223 231 10.1080/01443410303226

[B23] Rubin DB Estimating causal effects from large data sets using propensity scores Annals of Internal Medicine 1997 127 757 767 9382394 10.7326/0003-4819-127-8_part_2-199710151-00064

[B24] Kennedy P A guide to econometrics 1996 3 Cambridge: MIT Press

[B25] Rosenbaum PR Rubin DB The central role of the propensity score in observational studies for causal effects Biometrika 1983 70 1 41 55 10.1093/biomet/70.1.41

[B26] Heckman JJ Ichimura H Todd P Matching as an econometric evaluation estimator Review of Economic Studies 1998 65 261 294 10.1111/1467-937X.00044

[B27] Stecker PM Fuchs LS Fuchs D Using curriculum-based measurement to improve student achievement: Review of research Psychology in the Schools 2005 42 795 819 10.1002/pits.20113

[B28] Lipsey MW Design Sensitivity: Statistical Power for Experimental Research 1990 Newbury Park, CA: Sage

